# PCSK-9 inhibitors: a new direction for the future treatment of ischemic stroke

**DOI:** 10.3389/fphar.2023.1327185

**Published:** 2024-01-11

**Authors:** Lin Zhou, Hongyu Zhang, Shuyi Wang, Hong Zhao, Yongnan Li, Juqian Han, Hongxu Zhang, Xiaoyuan Li, Zhengyi Qu

**Affiliations:** ^1^ Department of Neurology, The Fourth Affiliated Hospital of Harbin Medical University, Harbin, China; ^2^ Department of Neurosurgery, The Fourth Affiliated Hospital of Harbin Medical University, Harbin, China; ^3^ State Key Laboratory of Ophthalmology, Zhongshan Ophthalmic Center, Guangdong Provincial Key Laboratory of Ophthalmology and Visual Science, Sun Yat-sen University, Guangzhou, China

**Keywords:** acute ischemic stroke, stroke, PCSK9 inhibitor, hyperlipidemia, platelets, atherosclerosis

## Abstract

Ischemic stroke, the most prevalent and serious manifestation of cerebrovascular disease, is the main cause of neurological problems that require hospitalization, resulting in disability and death worldwide. Currently, clinical practice focuses on the effective management of blood lipids as a crucial approach to preventing and treating ischemic stroke. In recent years, a great breakthrough in ischemic stroke treatment has been witnessed with the emergence and use of a novel lipid-lowering medication, Proprotein convertase subtilisin kexin type 9 (PCSK9) inhibitor. And its remarkable potential for reducing the occurrence of ischemic stroke is being acknowledged. This article aims to provide a comprehensive review, encompassing the association between PCSK9 and the heightened risk of ischemic stroke, the mechanisms, and the extensive evidence supporting the proven efficacy of PCSK9 inhibitors in clinical practice. Through this present study, we can gain deeper insights into the utilization and impact of PCSK9 inhibitors in treating ischemic stroke.

## 1 Introduction

Ischemic stroke is a neurological disorder caused by focal cerebral, spinal cord or retinal infarction (Sacco et al., 2013). According to guidelines reported by the American Heart Association and American Stroke Association in 2021, about 795,000 people experience a stroke each year in the United States, of which 87% are ischemic stroke and 185,000 are recurrent (Kleindorfer et al., 2021). The causes of ischemic stroke can be classified as follows: cardiogenic embolism, atherosclerosis of large vessels, blockage of minor vessels, as well as other definite and unidentified causes (Adams et al., 1993). Among these, lipid levels, degree of atherosclerosis, and high platelet activity are the main risk factors for ischemic stroke.

Proprotein convertase subtilisin kexin type 9 (PCSK9) has recently garnered significant attention as a target for increasing low-density lipoprotein cholesterol (LDL-C) levels in patients with hyperlipidemia. In many recent related studies, PCSK9 is closely related to atherosclerosis, platelet aggregation, thrombogenesis, and vascular aging. With the birth of PCSK9 inhibitors and their gradually popularized use, their benefits in the therapy of stroke have been progressively discovered. The aim of this review is to expound on the mechanism by which PCSK9 increases the risk of ischemic stroke as well as the efficacy and safety of PCSK9 inhibitors in the treatment of stroke. It also suggests the future directions of this therapy in stroke treatment and prevention.

## 2 Roles of PCSK9 in different ischemic stroke-related risk factors

### 2.1 Role of PSCK9 in lipid homeostasis

**FIGURE 1:**
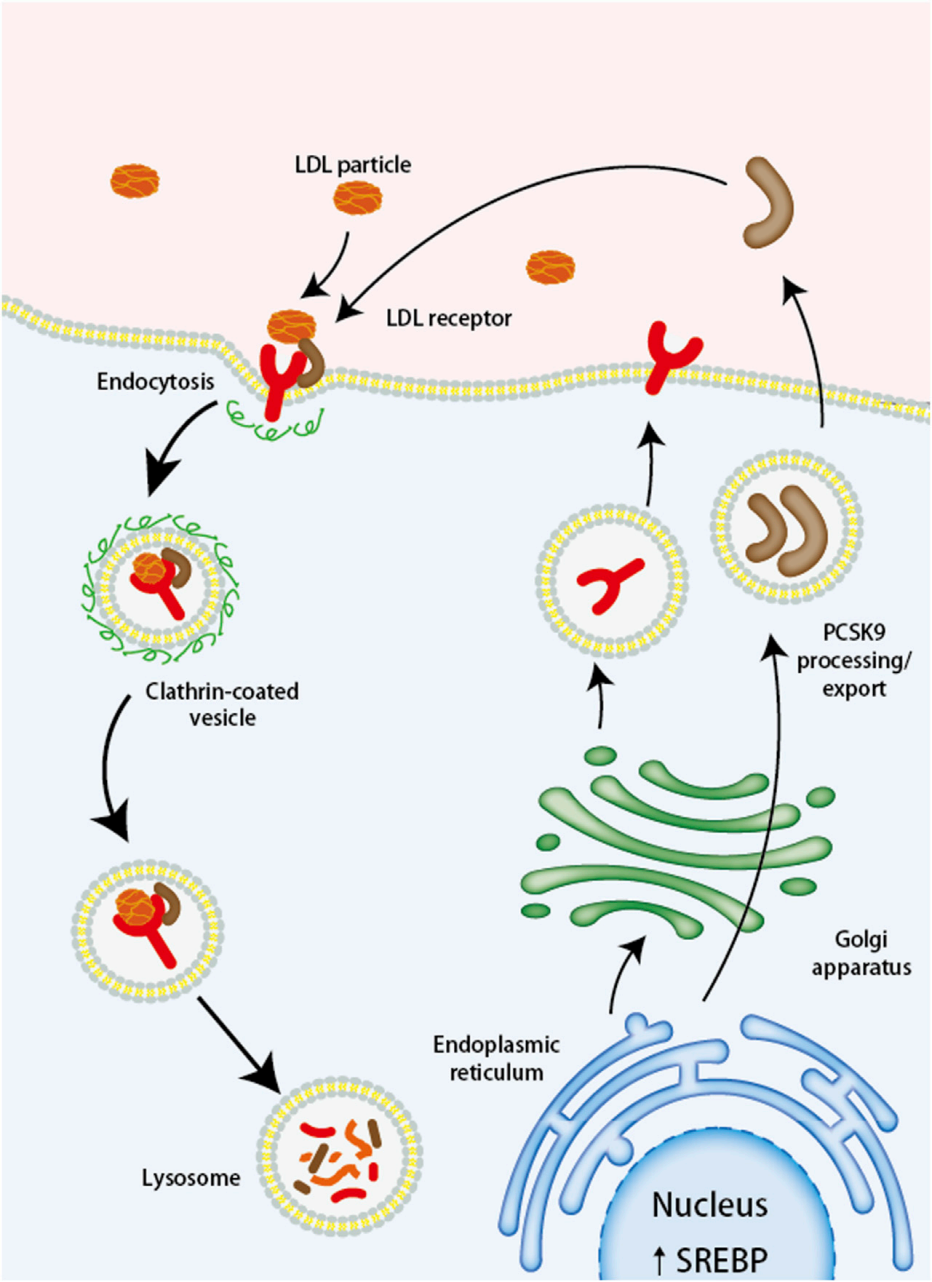
The Mechanism of PCSK9 Mediated LDL Receptor Degradation. LDL, low-density lipoprotein; LDL-R, low-density lipoprotein receptor; PCSK9, proprotein convertase subtilisin/kexin type 9; SREBP, sterol regulatory element binding protein.

PCSK9 belongs to the chymosin serine protease mainly synthesized and released by the liver, small intestine, and kidneys (Seidah and Prat, 2012), playing an important role in maintaining the homeostasis of cholesterol. Lots of studies have shown that PCSK9 increases the level of LDL-C in the body by inhibiting the recirculation of the low-density lipoprotein receptor (LDL-R) (Ilut et al., 2022). When LDL-R recognizes and binds to LDL-C, it forms a complex that enters the cell through a lattice-protein heavy chain-coated vesicle and is internalized into endosomes. The acidic environment causes LDL-R to metastasize and dissociate, and LDL-R is recycled to the cell surface, while LDL-C is sent to lysosomes for degradation, and its cholesterol is recycled and stored in the cell. Contrastingly, PCSK9 first binds to LDL-R, forming a complex with LDL-C and LDL-R that enters the cell via vesicles. This complex, however, cannot dissociate in the acidic environment of the endosomes upon internalization. Instead, they are transported together into the lysosome for degradation (Seidah et al., 2014) (Figure 1). In addition, PCSK9 can also enhance the degradation of LDL-R through intracellular pathways or bind LDL-R directly at the cell surface through extracellular pathways (Poirier et al., 2009), both of which contribute to the failure of LDL-C degradation in the body.

Several studies indicate that PCSK9 can degrade not only LDLR but also low-density lipoprotein receptor-related protein 1 (LRP-1), the very low-density lipoprotein receptor (VLDLR), and the apolipoprotein E2 receptor (ApoER2) (Poirier et al., 2008; Canuel et al., 2013). Furthermore, PCSK9 can directly increase the production of hepatic lipids and lipoprotein through ApoE and LDLR-dependent mechanisms (Tavori et al., 2016).

### 2.2 Effects of PCSK9 on atherosclerosis

Atherosclerosis is a lipoprotein-driven disease that causes plaque formation in the arterial tree at specific locations through intimal inflammation, necrosis, fibrosis and calcification (Bentzon et al., 2014). The accumulation of cholesterol in the arterial intima is mainly due to the oxidative modification of accumulated low-density lipoprotein cholesterol (LDL) by monocytes and macrophages to form aggregated oxidized low-density lipoprotein (ox-LDL) (Llorente-Cortes et al., 2004; Costales et al., 2015). The accumulation of ox-LDL in the arterial wall occurs through the high expression of scavenger receptor A (SRA), cluster of differentiation 36 (CD36), and lectin-like oxidized LDL receptor 1 (LOX-1) under different inflammatory stimulations. LOX-1 expression on macrophages, thereby affecting ox-LDL uptake and thus contributing to the atherogenic process. One trial showed that a 2- to 5-fold increase in LOX-1, SRA, and CD36 expression and a 5-fold increase in ox-LDL uptake were observed after treatment of TNF-α-initiated macrophages with recombinant mouse PCSK9 (Ding et al., 2018). Additionally, there is positive feedback between PCSK9 and LOX-1 in vascular smooth muscle cells (VSMCs). Activation of LOX-1 stimulates PCSK9 expression, while PCSK9 promotes LOX-1 expression and ox-LDL uptake, inducing an inflammation state. Whereas, the pro-inflammatory stimulators, including tumor necrosis factor-α (TNF-α) and lipopolysaccharide (LPS), increase PCSK9 expression in VSMCs and vascular endothelial cells in an inflammatory environment (Ding et al., 2020), leading to increased accumulation of lipoproteins in the artery wall and greatly promoting the onset of atherosclerosis. Therefore, PCSK9 was shown to promote ox-LDL-induced atherosclerosis endothelial cell apoptosis via the JNK/p38 MAPK pathway (Li et al., 2017), which in turn reduced the stability of atherosclerotic plaques (Shan et al., 2021). The rupture of unstable atherosclerotic plaques, leading to a subsequent thrombus, may result in serious clinical complications such as myocardial infarction or ischemic stroke (Basatemur et al., 2019). Additionally, during the development of atherosclerotic plaques in atherogenesis, macrophages and smooth muscle cells produce ApoE, which reduces intracellular lipoprotein accumulation, inhibits the formation of foam cells, and promotes the anti-inflammatory phenotype of macrophages through ApoER2 (Bai et al., 2018). Whereas PCSK9 can reduce ApoER2 expression (Poirier et al., 2008), the protective effect exerted by ApoE is greatly attenuated.

Serum PCSK9 was first demonstrated to be associated with cardiovascular disease (CVD) in a 60-year-old population without CVD, and even when established CVD risk variables were modulated, this association persisted (Leander et al., 2016). Another study demonstrated that serum PCSK9 levels independently predicted carotid intima-medial wall thickness, even after adjusting for factors like gender, hypertension, smoking status, Homeostasis Model Assessment (HOMA) score, obesity, LDL-C, lipoprotein (a), and inflammatory markers. This suggests that PCSK9 levels might contribute to an increased risk of carotid atherosclerosis independently of systemic lipid alterations without systemic lipid changes (Chan et al., 2016).

In general, PCSK9 can affect the progression of atherosclerosis by raising blood lipids and atherosclerotic plaque accumulation (Denis et al., 2012). Therefore, anti-PCSK9 therapy is very beneficial for cardiovascular disease, cerebrovascular disease, and other vascular diseases caused by atherosclerosis.

### 2.3 Effects of PCSK9 on platelets and thrombosis

Embolism is the most common cause of ischemic stroke (Feske, 2021). Thrombosis is strictly dependent on platelet adhesion, activation, and aggregation (Denis and Wagner, 2007). In a prospective, single-center cohort study in 2017, a direct association of PCSK9 with 11-dehydrothromboxane B2 (11-dh-TxB_2_) was observed by using an enzyme-linked immunosorbent assay to determine urinary excretion of 11-dh-TxB_2_, suggesting that PCSK9 may be directly associated with platelet activation (Pastori et al., 2017). In an experiment conducted in the same year that compared PCSK9-deficient mice to normal mice, it was found that 48 h after ligation of the inferior vena cava, 60% of mice in the control group developed significant thrombus, while only 5% of PCSK9-deficient mice developed thrombus with a smaller weight and length of the thrombus (Wang et al., 2017). In a prospective observational study, a direct association between elevated PCSK9 serum levels and higher platelet reactivity was found, and it predicts that elevated PCSK9 serum levels will be a predictor of ischemic events after percutaneous coronary intervention (PCI) in acute coronary syndrome (ACS) patients in the future (Navarese et al., 2017). The result from a cross-sectional study of 89 participants showed that a significant correlation was found between adenosine diphosphate (ADP)-induced maximum aggregation rate (MAR) and PCSK9, as well as serum total cholesterol (TC), LDL-C, and platelet (PLT), and between PCSK9 and arachidonic acid (AA) induced platelet. In addition, multiple regression analysis showed that PCSK9 had an independent effect on ADP-induced maximum aggregation rate after controlling for the effects of TC, LDL-C, PLT, gender, and smoking (Wang et al., 2022). These experiments suggest that PCSK9 can act as an independent factor associated with increased platelet hyperactivity and accelerated thrombosis.

Indeed, on the one hand, elevated PCSK9 levels can indirectly activate platelets by reducing the depletion of lipoproteins, which in turn can promote platelet hyperactivation (Ding et al., 2022). Specifically, LOX-1 in lipoproteins facilitates the activation of endothelial cells and platelets by ox-LDL. This process triggers the activation of MAPK extracellular signal-regulated kinase 5 (ERK5), which plays a pivotal role in regulating the platelet CD36 signaling pathway, thereby promoting thrombosis (Yang et al., 2017). Additionally, coagulation factor VIII is crucial in thrombus formation. LDL-R aids in clearing coagulation factor VIII, and PCSK9’s role in lowering LDL-R can lead to diminished *in vivo* clearance of factor VIII, consequently increasing thrombus formation (Paciullo et al., 2019). On the other hand, PCSK9 directly enhances platelet activation and thrombosis *in vivo* by binding to the CD36 receptor in platelets independently of LDL-related pathways. In a study of myocardial infarction mice model injured by Fecl_3_, PCSK9 binds platelet CD36, which activates Src kinase and MAPK/EMRK5 and MAPK/JNK. This activation leads to an increase in reactive oxygen species production, which is followed by synergistic downstream signaling induced by activation of the p38MAPK/cytoplasmic phospholipase A2/cyclooxygenase 1/thrombospondin A2 signaling pathway downstream of CD36 that converts thromboxane synthetase activity to thromboxane A2 (TX A2), which binds to various platelet agonist receptors, and then activates integrin αIIbβ3 (Qi et al., 2021). Integrin αIIbβ3 alters platelet conformation, leading to platelet proliferation, aggregation, and thrombosis (Coller, 2015; van den Kerkhof et al., 2021). Encouragingly, the trial also found that Evolocumab inhibited the enhancement of platelet activity by PCSK9 (Qi et al., 2021), indicating that patients at risk of stroke due to overactive platelets now have a new treatment option.

## 3 Clinical application of PCSK9 inhibitors

### 3.1 Classic PCSK9 inhibitors

**FIGURE 2:**
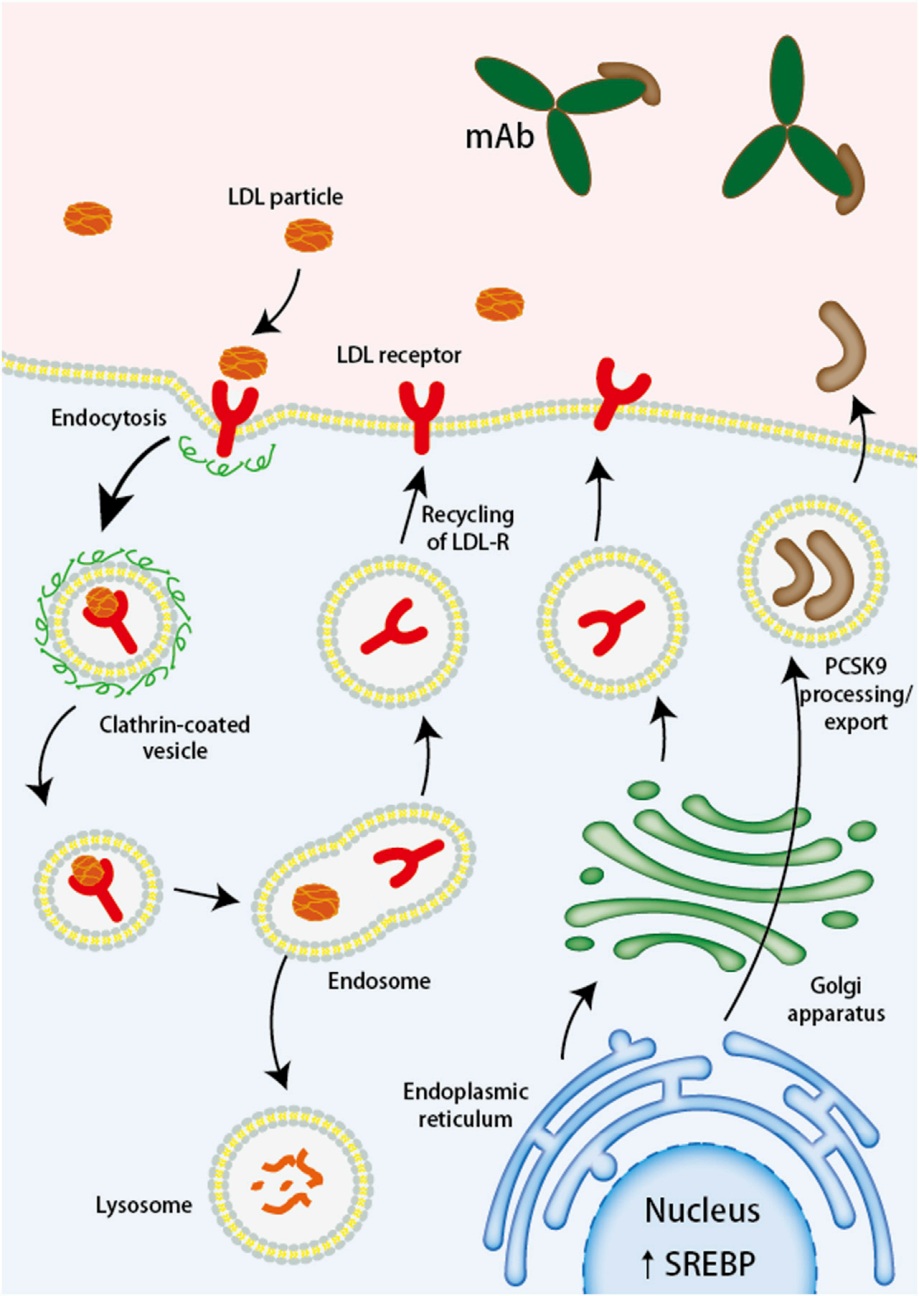
Monoclonal antibodies targeting PCSK9 bind to PCSK9, preventing the degradation of LDL receptors. LDL, low-density lipoprotein; LDL-R, low-density lipoprotein receptor; mAb, monoclonal antibody; PCSK9, proprotein convertase subtilisin/kexin type 9; siRNA, small interfering RNA; SREBP, sterol regulatory element binding protein.

Evolocumab and Alirocumab are PCSK9 inhibitors currently approved for use and are fully human monoclonal antibodies (mAbs) (Pirillo et al., 2021). The mAbs bind to PCSK9 and block the binding site of LDL-R, which inhibits the interaction between PCSK9 and LDL-R, and increases the expression of LDL-R on the cell surface so that more LDL-C can bind to LDL-R and be degraded by lysosomes thus reducing blood lipid levels *in vivo* (Kasichayanula et al., 2018; Roth and Davidson, 2018) (Figure 2). Under the influence of the lack of lipids and PCSK9, atherosclerotic disease is delayed or even improved, and the formation of thrombosis in the body is reduced, which not only dramatically reduces the risk of ischemic stroke but also has important implications for the prognosis of ischemic stroke. Alirocumab, the first PCSK-9 inhibitor approved by the FDA, is indicated for use in patients with familial hypercholesterolemia or those who require additional lowering of LDL cholesterol (Merćep et al., 2022). Through an investigation comparison of 18,924 patients with acute coronary syndromes, the ODYSSEY trial examines the effectiveness and safety of Alirocumab in lowering LDL-C levels and CV events. The primary efficacy endpoint is coronary heart disease, non-fatal myocardial infarction, fatal or non-fatal ischemic stroke, or unstable angina requiring hospitalization. The results showed a 62% reduction in LDL-C at week 78 in the Alirocumab group compared with the placebo group (Schwartz et al., 2018). To evaluate Alirocumab’s impact on ischemic stroke, a sub-study of the ODYSSEY trial was conducted with patients having recent ACS and dyslipidemia on intensive statin therapy. The study compared Alirocumab with placebo and found no increased risk of hemorrhagic stroke (Jukema et al., 2019).

Evolocumab, approved by the FDA in 2015, is indicated for patients with familial hypercholesterolemia or atherosclerotic cardiovascular disease who require additional lowering of LDL cholesterol (Attipoe-Dorcoo et al., 2021). The findings from the Further Cardiovascular Outcomes Research with PCSK9 Inhibition in subjects with Elevated Risk (FOURIER) trial is an experiment to observe the efficacy and safety of statin therapy in combination with Evolocumab. In a comparison of 27,564 patients with atherosclerotic cardiovascular disease who received statin treatment, the primary outcome was cardiovascular death, myocardial infarction, stroke, hospitalization for unstable angina, or coronary revascularization as the main efficacy endpoint. The key secondary efficacy endpoint was cardiovascular death, myocardial infarction, or stroke (Sabatine et al., 2016). When compared to the placebo group at week 48, the results demonstrated a 15% reduction in the risk of the primary endpoint and a 20% reduction in the hazard of an ischemic stroke (Sabatine et al., 2017a). A pre-analysis of cerebrovascular events in patients with prior stroke in FOURIER showed significant efficacy of Evolocumab in patients with established atherosclerosis are at risk for all types of stroke, including ischemic stroke, hemorrhagic stroke, or transient ischemic attack (TIA) in combination with post-stroke dependency (Giugliano et al., 2020). Evolocumab safely reduced the concentration of blood LDL-C and reduced the risk of recurrent ischemic stroke and TIA in the future (Giugliano et al., 2020). A 52-week placebo-controlled trial of Evolocumab in the treatment of hyperlipidemia showed a 55.7% ± 4.2% decrease in LDL-C levels compared to placebo in patients treated solely with dietary therapy (Blom et al., 2014). In addition, the Goal Achievement After Utilizing an AntiPCSK9 Antibody in Statin Intolerant Subjects 2 (GAUSS-2) and GAUSS-3 studies showed that Evolocumab significantly reduced the level of LDL-C without using statins and had a more significant effect on reducing lipid than Ezetimibe (Stroes et al., 2014; Nissen et al., 2016). PCSK9 inhibitors decrease the level of cholesterol when combined with other lipid-lowering medications like statins and Ezetimibe. Patients who are receiving lipid-lowering drugs may experience better lipid-lowering effects using PCSK9 inhibitors (Gil-Nunez et al., 2022).

A meta-analysis found an efficacy gap between these two mAbs, with a 2-week or monthly dose of Evolocumab being 14%–20% more effective in reducing the level of LDL-C than a 2-week, or monthly dose of Alirocumab (Toth et al., 2017b). Therefore, Evolocumab is generally preferred for treatment.

Besides their significant effects on lipids and ischemic stroke, PCSK9 inhibitors can also mitigate risk factors for ischemic stroke, particularly in the treatment of atherosclerosis. Recently, several studies have demonstrated that Evolocumab and Alirocumab, respectively, can increase the thickness of the fibrous cap in the early stage and reduce the regression of lipid-rich plaques, ultimately improving plaque morphology (Kuhnast et al., 2014; Yano et al., 2020). The results of a Global Assessment of Plaque Regression With a PCSK9 Antibody as Measured by Intravascular Ultrasound (GLAGOV) randomized clinical trial designed to study the effect of Evolocumab on the progression of coronary atherosclerosis in patients treated with statins showed that regression of coronary atherosclerosis occurred in 47% of people in the placebo group, and in 64% of patients in the therapy group receiving the combination of statins and Evolocumab with a substantial decrease in the percentage of atherosclerotic plaque volume after 76 weeks of subcutaneous Evolocumab injections (Nicholls et al., 2016). Of concern, in a 2021 trial, two young patients with pure-sibling familial hypercholesterolemia (12 years old and 16 years old) experienced almost complete regression of plaque after intensive lipid-lowering with PCSK9 inhibitors in combination with other lipid-lowering agents (Reeskamp et al., 2021). This finding suggests that high-intensity lipid lowering in the early stages may not only affect coronary plaque formation but may even completely restore atherosclerosis. Although many PCSK9 inhibitors have been found to improve atherosclerotic plaque, more time is needed to observe the effect of this improvement.

### 3.2 Inclisiran inhibits PCSK9

Inclisiran is a chemically modified double-stranded small interfering RNA that inhibits PCSK9 synthesis by specifically targeting PCSK9 mRNA in hepatocytes (Ray et al., 2018). Compared to the two monoclonal antibodies mentioned above, Inclisiran only requires dosing at the initial baseline and every 6 months after the 3-month dose, which greatly improves patient adherence to treatment (Pirillo and Catapano, 2022).

The primary outcome of the phase 2 Inclisiran study ORION-1 was the difference between baseline and day 180 LDL-C values after using Incisiran. The results indicate that PCSK9 and LDL cholesterol levels decreased in patients using Incisiran (Ray et al., 2017). The patients with atherosclerotic cardiovascular disease or an atherosclerotic cardiovascular disease risk equivalent (ORION-11) trial is a phase 3 trial of Inclisiran. The primary endpoints were the change in cholesterol levels from baseline to day 510 and the change in cholesterol levels from baseline to time-adjusted levels after 90 days to day 540, and the results found a decrease in PCSK9 levels compared to placebo in almost all patients receiving Inclisiran (Ray et al., 2020). In further analysis of the ORION-11 trial, patients were found to significantly reduce atherogenic lipoprotein levels by using Inclisiran twice a year (Ray et al., 2022). Furthermore, Inclisiran has been shown to prevent or substantially slow the formation of atherosclerotic plaques (Pirillo and Catapano, 2022). In terms of safety, no clinical adverse reactions have been clearly associated with Inclisiran, apart from the occurrence of injection site reactions (Ray et al., 2017; Ray et al., 2018; Raal et al., 2020; Ray et al., 2020). Unfortunately, so far, no clinical trials have reported definitive efficacy of Inclisiran for ischemic stroke, except for the treatment impact of Inclisiran on lipids and atherosclerosis, either of which are indicators of risk for ischemic stroke.

## 4 Safety of PCSK9 inhibitors

The ODYSSEY trial reported similar rates of adverse events (neurocognitive events, new-onset diabetes, hemorrhagic stroke) in the Alirocumab and placebo groups, with no increase in the incidence of these adverse events. Patients in the Alirocumab group experience injection site reactions (pruritus, redness, or swelling), but such injection reactions are usually mild and self-limiting (Schwartz et al., 2018). Similarly, there is no significant difference in the incidence of adverse events or serious adverse events reported in the FOURIER study between Evolocumab and placebo (Sabatine et al., 2017b). Some studies suggest that decreased LDL-C levels lead to an increase in neurocognitive events. A Cambridge Neuropsychological Test Automated Battery (EBBINGHAUS) study studied 1,974 patients in the FOURIER trial and found that there are no significant differences in cognitive function between patients treated with Evolocumab and those treated with placebo (Giugliano et al., 2017a). Further exploratory analysis of this trial concluded that the levels of LDL-C are not associated with neurocognition (Giugliano et al., 2017b). However, the sample size of this trial is limited, and it is necessary to carefully study the effects of the PCSK9 monoclonal antibody on neurocognitive function in a sufficiently large patient population.

In a pooled analysis of the ODYSSEY Phase III trial, Alirocumab was found to have no effect on glycemic or safety parameters (Leiter et al., 2018). Further analysis of the FOURIER trial shows that Evolocumab did not increase the risk of new-onset diabetes or worsen glycemia (Sabatine, et al., 2017b). A similar 52-week placebo-controlled trial of Evolocumab, another PCSK-9 inhibitor, is also approved for reducing LDL cholesterol levels, the DESCARTES study, found that Evolocumab showed satisfactory safety and efficacy at 52 weeks in patients with or without dysglycemia or metabolic syndrome. Additionally, there were no variations in glycemic metrics between individuals receiving Evolocumab and those receiving a placebo in the subgroup of glycemic parameters (Blom et al., 2017).

Moreover, several studies have found no close correlation between the effects of PCSK9 inhibitors on muscle adverse effects, liver function metabolism, and endocrine function (Toth et al., 2017a; Koren et al., 2017; Roth, 2018), suggesting that PCSK9 inhibitors are clinically applicable to the majority of patients and produce few side effects.

Two mAbs, Alirocumab and Evolocumab bind to PCSK9 with high specificity, alleviating concerns about drug-drug interactions (Roth and Davidson, 2018). In addition, mAbs are large molecular proteins (Davidson and Roth, 2018), and have difficulty crossing the blood-brain barrier. Both of these mAbs are metabolized by the reticuloendothelial system and are finally broken down into small peptides and amino acids. Since they are not metabolized by the liver and kidneys, they alleviate the concerns of patients with liver and kidney disease (Roth and Davidson, 2018).

Alirocumab and Evolocumab have been in clinical use for several years, and their potent lipid-lowering effects and ability to reduce the risk of vascular events have been recognized, but their injection site response still needs to be improved. However, these two mAbs need to be injected every 2 weeks, which greatly reduces patient compliance (Lamb, 2021). Inclisiran, approved for clinical use in the European Union in 2020, has improved patient compliance by being administered at a frequency of once every 6 months. Since the clinical use of Inclisiran is relatively short, its efficacy and safety remain to be investigated, especially since Inclisiran targets hepatocytes, and therefore, its effect on liver function deserves our attention in the future. Tables 1, 2 summarize the study characteristics of some clinical trials of PCSK9 inhibitors, their effects on stroke and stroke risk factors, and the unusual adverse effects.

**TABLE 1:** Baseline characteristics of the trial for PCSK9 inhibitors.

Trial	Year	Number of samples	Age	Male proportion %	Types of PCSK9 inhibitors	Dose usage	Control	Follow-up time
FOURIER (Sabatine et al., 2017a)	2017	27,564	62.5 ± 9.1	75.4	Evolocumab	140 mg/2 weeks Or 420 mg/4 weeks	Placebo	52 weeks
ODYSSEY OUTCOMES (Schwartz et al., 2018)	2018	18,924	58.5 ± 9.3	74.8	Alirocumab	75 mg/2 weeks	Placebo	146 weeks
ORION-10 (Ray et al., 2020)	2020	1,561	66.4 ± 8.9	69.4	Inclisiran	284 mg/day 1, day 90, and every 6 months	Placebo	78 weeks
ORION-11 (Ray et al., 2020)	2020	1,617	64.8 ± 8.3	71.5	Inclisiran	284 mg/day 1, day 90, and every 6 months	Placebo	78 weeks

**TABLE 2:** Results reports on PCSK9 inhibitors.

Trial	Brain stroke		Ischemic stroke		LDL-C level compared to baseline		Adverse event
	PCSK9 inhibitors	Control	PCSK9 inhibitors	Control	PCSK9 inhibitors	Control	
FOURIER (Sabatine et al., 2017a)	207	251	171	226	Decreased by 56 mg/dL	Decreased about 2 mg/dL	The injection site reaction of Evolocumab is more common, and there is no significant difference in the rest
ODYSSEY OUTCOMES (Schwartz et al., 2018)	123	170	111	152	Significantly reduce	Slight increase	The injection site reaction of Alirocumab is more common, and there is no significant difference in the rest
ORION-10 (Ray et al., 2020)	11	7	7	4	Decreased by 51.3%	Increase by 4.0%	The injection site reaction of Inclisiran is more common, and there is no significant difference in the rest
ORION-11 (Ray et al., 2020)	2	8	1	3	Decreased by 45.8%	Increase by 3.4%	The injection site reaction of Inclisiran is more common, and there is no significant difference in the rest

In recent years, it has been observed that PCSK9 is dependent on adenylate cyclase-associated protein 1 (CAP1) when binding to LDL-R, which has been proposed as a necessary condition for PCSK9 to degrade LDL-R (Jang et al., 2020). Therefore, lipid-lowering could be achieved by eliminating CAP1 in the future. It is of particular concern that PCSK9 inhibitors can antagonize the enhanced effect of PCSK9 on platelet activity, so it is worth considering whether to use PCSK9 inhibitors directly to alter the prognosis of patients with ischemic stroke with increased platelet activity. The mechanism of increased platelet activity of PCSK9 is not yet fully understood, and more trials can be conducted to further understand the mechanism in order to improve PCSK9 inhibitors and make them more targeted in the treatment of ischemic stroke.

Moreover, while the application of PCSK9 inhibitors in ischemic stroke primarily pertains to the chronic phase, Alirocumab and Evolocumab have shown the capability to rapidly reduce PCSK9 levels within hours, potentially diminishing the associated risk of ischemic stroke. Therefore, it is necessary to evaluate the efficacy of PCSK9 inhibitors in the acute phase of ischemic stroke in the future. Patients with a TIA have a higher risk of early stroke than other suspected risks, with 10%–15% of patients having a stroke within 3 months and 50% of them having a stroke within 48 h after the onset of TIA (Easton et al., 2009). Therefore, TIA is again an early warning event of stroke. Thus, it is necessary to use PCSK9 inhibitors to treat the prognosis of TIA patients.

## 5 Some ongoing clinical trials of PCSK9 inhibitors

Several clinical trials of PCSK9 inhibitors in patients with ischemic stroke are ongoing, some studies are looking at the effect of PCSK9 inhibitors on acute stroke, and some studies are looking at the effect of PCSK9 inhibitors on intracranial atherosclerotic plaques. Ongoing clinical trials are shown in Table 3. It is believed that these trials will provide important information for the treatment of ischemic stroke, and there are currently no trials for Inclisiran in ischemic stroke.

**TABLE 3:** Ongoing Trial on PCSK9 inhibitors.

Study name	Number	Using medication	Experimental location	Aim
RMeng	NCT06134635	Evolocumab Statins	China	The early impact of Evolocumab on patients with acute ischemic stroke (AIS) in China
TOPICAL-MRI	NCT05001984	Alirocumab	China	This study will evaluate whether low-density lipoprotein (LDL-C) lowering with alirocumab results in greater change from baseline in intracranial atherosclerotic plaque at week 26 than control in adults with acute ischemic stroke from intracranial atherosclerosis taking lipid lowering therapy
PCSK9_001	NCT06083961	Alirocumab	Seoul, Korea	The goal of this clinical trial is to test the effect of proprotein convertase subtilisin/kexin type 9 (PCSK9 inhibitors) in acute ischemic stroke patients associated with atherosclerosis by investigating
00121763	NCT04573777	Evolocumab	United States	The aim of this study was to investigate the potential mechanism by which PCSK9 inhibition reduces the occurrence of ischemic stroke, and the researchers hypothesized that PCSK9 inhibition reduces the incidence of stroke by reducing atherosclerotic plaque, which may be of particular benefit to patients with intracranial atherosclerosis, who are at the highest risk of recurrent stroke of all stroke mechanisms
sICASBLM	NCT05397405	PCSK9 inhibitor	China	sICASBLM is a prospective controlled trial, to assess the impact of improving blood lipid management on clinical outcome of moderate to severe symptomatic intracranial atherosclerotic stenosis patients (LDL-C > 1.8 mmol/L) without endovascular therapy

## 6 Conclusion

With the in-depth study of PCSK9, more scholars have discovered its mechanism of raising blood lipids, accelerating the formation of atherosclerosis and participating in the formation of thrombosis. In addition, the development of PCSK9 inhibitors has become an important means to treat hyperlipidemia and atherosclerosis. Several recent trials have shown that PCSK9 inhibitors reduce platelet activity and that thrombosis significantly reduces the risk of ischemic stroke. Current clinical trials have confirmed the use of PCSK9 inhibitors to treat and prevent stroke. PSCK9 inhibitors will become an important drug for the treatment of ischemic stroke in the future. Finally, it is worth keeping an eye on the ongoing clinical trials of PCSK9 inhibitors to gain insight into the efficacy of these inhibitors and the optimal dose to use. In the future, we should also conduct clinical trials of PCSK9 inhibitors in patients with TIA (disease events prone to progression to ischemic stroke) to develop their broader adaptation.
